# Solution-Processed Pure Blue Thermally Activated Delayed Fluorescence Emitter Organic Light-Emitting Diodes With Narrowband Emission

**DOI:** 10.3389/fchem.2021.691172

**Published:** 2021-05-20

**Authors:** Ting Xu, Xiao Liang, Guohua Xie

**Affiliations:** ^1^ Shenzhen Key Laboratory of Polymer Science and Technology, College of Materials Science and Engineering, Shenzhen University, Shenzhen, China; ^2^ Key Laboratory of Optoelectronic Devices and Systems of Ministry of Education and Guangdong Province, College of Physics and Optoelectronic Engineering, Shenzhen University, Shenzhen, China; ^3^ State Key Laboratory of Surface Physics and Department of Physics, Fudan University, Shanghai, China; ^4^ Jiangsu Key Laboratory for Carbon-Based Functional Materials and Devices, Soochow University, Suzhou, China; ^5^ State Key Laboratory of Coordination Chemistry, Jiangsu Key Laboratory of Advanced Organic Materials, School of Chemistry and Chemical Engineering, Nanjing University, Nanjing, China; ^6^ Department of Chemistry and Hubei Key Lab on Organic and Polymeric Optoelectronic Materials, Wuhan University, Wuhan, China

**Keywords:** organic light-emitting diodes, thermally activated delayed fluorescence, solution process, narrow bandwidth, pure blue

## Abstract

There is a need to satisfy the high color purity requirement of display technology with a simply fabricated process. Herein, solution-processed blue thermally activated delayed fluorescence organic light-emitting diodes (OLEDs) with a narrow spectrum with a full width at half maximum (FWHM) of 32 nm and y color coordinate below 0.2 are demonstrated by employing a molecule containing boron and nitrogen atoms (TBN-TPA) as the guest emitter in the emissive layer. The opposite resonance positions of B-N atoms of TBN-TPA endows a multi-resonance effect, leading to high color purity.

## Introduction

Solution-processed organic light-emitting diodes (s-OLEDs) are regarded as one of the most fascinating and competitive technologies for large-area and low-cost display panels and solid-state lighting sources (Müller et al., 2003; So et al., 2008; Zhu et al., 2011; Sasabe and Kido, 2013; Zheng et al., 2013; Xu et al., 2017a; Xu et al., 2019; Wang et al., 2020; Xu et al., 2021). Modern electronic products can be easily manufactured by ink-jet printing or ‘roll-to-roll’ coating methods, akin to how newspapers are produced. However, the current state-of-the-art OLEDs rely on physical vapor deposition, which leads to high manufacturing costs and energy consumption (Kololuoma et al., 2004; Mauthner et al., 2008; Sandström et al., 2012; Xu et al., 2016). The invention and application of thermally activated delayed fluorescence (TADF) compounds as the emitters without precious metals (e.g., iridium, platinum, rhenium, etc.), further facilitate more cost-effective OLED technology (Uoyama et al., 2012; Huang et al., 2018; Xu et al., 2018). Blue color plays an important role as one of the three primary colors of OLEDs. A novel concept for multi-resonance TADF (MR-TADF) was proposed by Hatakeyama et al. in 2016. The reported TADF emitters show particular HOMO and LUMO distributions due to the rigid framework of boron and nitrogen (B-N) atoms’ array, leading to the MR effect. This MR effect enhances the oscillating strength between S_1_ and S_0_, generating a narrow full-width-at-half-maximum (FWHM) of 28 nm, showing unexpected high color purity in TADF species (Hatakeyama et al., 2016). Then highly efficient blue MR-TADF molecules were developed, showing an extremely high external quantum efficiency (EQE) of up to 34.4% and a narrow FWHM of 14 nm (Kondo et al., 2019). However, blue MR-TADF materials with high color purity are rarely applied in s-OLED. Therefore, it is attractive to investigate blue MR-TADF materials aiming for a solution process.

In this paper, solution-processed blue TADF OLEDs with a narrow bandwidth have been demonstrated by applying a molecule TBN-TPA (Liang et al., 2018) containing boron and nitrogen (B-N) atoms. Our results provided new understanding of the electroluminescence of blue MR-TADF emitter, which would be beneficial to the development of solution-processed OLED technology for high-performance displays.

## Experiment Details

**FIGURE 1:**
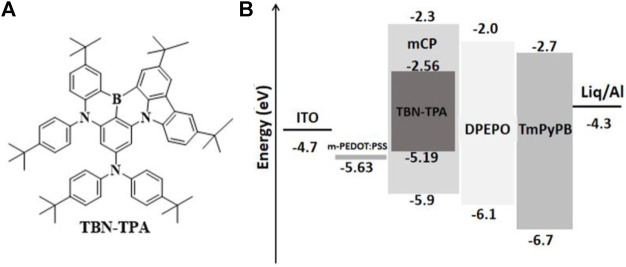
**(A)** The chemical structure of TBN-TPA. **(B)** Energy level diagrams of the devices.

The chemical structures of TBN-TPA and the energy level diagram of the device are exhibited in Figures 1A,B, respectively. TBN-TPA was synthesized according to the literature (Liang et al., 2018). 1,3-bis(9H-carbazol-9-yl)benzene (mCP), 8-hydroxyquinolinolatolithium (Liq), bis [2-(diphenyl-phosphino)phenyl]ether oxide (DPEPO), 1,3,5-tri [(3-pyridyl)-phen-3-yl] benzene (TmPyPB), and 8-hydroxyquino -linolatolithium (Liq) were purchased from Xi'an Polymer Light Technology Corporation and used as received.

All the devices were fabricated on the glass substrate patterned with the conducting indium-tin-oxide (ITO) anode with a sheet resistance lower than 20 Ω/square. Acetone and ethanol were consecutively used to clean the ITO substrates in an ultrasonic bath. The substrates were further dried with N_2_ flow. After 20 min of ultraviolet light-ozone treatment, a modified PEDOT: PSS (m-PEDOT:PSS) was spin-coated onto the ITO surface at 4,000 rpm (Xiang et al., 2019). Afterward, the substrate was baked at 120°C for 10 min in a glove box. TBN-TPA and mCP, respectively as the guest and host of the emissive material layer (EML), sufficiently dissolved in chlorobenzene solvent. Later, the corresponding EML was spin coated at 1,000 rpm and then accompanied with a 50°C baking process for 10 min. The corresponding functional materials and aluminum cathode were vacuum deposited step by step under 10^−5^ mbar. The actual device area defined by the crossover of the ITO anode and the Al cathode was 2 mm × 2.2 mm.

In this study, the OLEDs using the conventional mCP host were fabricated while TBN-TPA with the B-N core-structure containing a peripheral electron-donating carbazole unit was applied as the blue guest. The structure of the devices is ITO/m-PEDOT:PSS/TBN-TPA (5 wt%, 10 wt%, and 20 wt%): mCP/DPEPO (10 nm)/TmPyPB (50 nm)/Liq (1 nm)/Al (100 nm). The energy level diagram of the device is shown in Figure 1B. m-PEDOT:PSS acts as the hole injection layer (HIL) (Xiang et al., 2019). DPEPO serves as the exciton blocking layer with a high triplet energy level over 3.0 eV, which helps to confine the excitons in the emitting layer (Xu et al., 2017b). Liq and TmPyPB are used as the electron injection layer (EIL) and electron transporting layer (ETL), respectively.

The current density-voltage-luminance (J-V-L), the current efficiency-luminance -power efficiency (CE-L-PE) characteristics, the color coordinates, and the electroluminescence (EL) spectra of the devices were measured and recorded by a computer-controlled Keithley 2,400 Source Meter Unit and Photo Research PR735 spectrometer. All measurements were carried out at room temperature in ambient air.

## Results and Discussion

**FIGURE 2:**
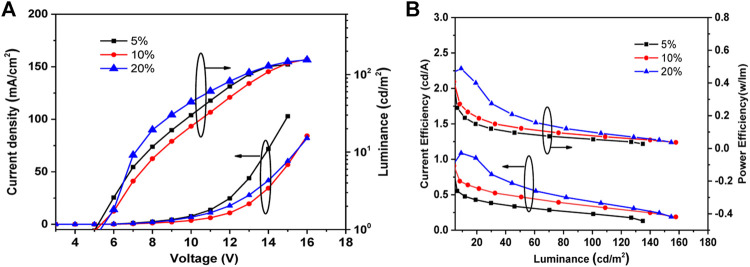
**(A)** Current density-voltage-luminance and **(B)** current efficiency-luminance-power efficiency characteristics of the devices.

The devices A, B, and C were fabricated and tested in one flow doping with different TBN-TPA concentrations i.e., 5 wt%, 10 wt%, and 20 wt%, respectively. The J-V-L and CE-L-PE characteristics are shown in Figures 2A,B, respectively. The device performances of the blue TADF OLEDs with different doping concentrations of TBN-TPA are summarized in Table 1.

**TABLE 1:** The EL characteristics of blue TADF OLEDs.

Device	Processing/Host	Vo_n_ ^a^ [V]	EL_peak_ [nm]	CE_max_ ^b^ [cd A^−1^]	FWHM^c^ [nm]	PE_max_ ^d^ [lm W^−1^]	EQE_max_ ^e^ [%]	CIE^f^ [x, y]
A	Solution/mCP	4.5	464	0.55	32	0.25	0.66	0.19, 0.14
B	Solution/mCP	4.8	464	0.91	32	0.41	1.03	0.19, 0.15
C	Solution/mCP	5.0	464	1.08	32	0.49	1.08	0.19, 0.19

a The turn-on voltage recorded at a brightness of 1 cd m^−2^.

b Maximum value ^2^ of current efficiency.

c Full-width-at-half-maximum of the EL spectrum.

d Power efficiency.

e External quantum efficiency.

f Commission Internationale de l’Eclairage (CIE) coordinates.

The turn-on voltage of the s-OLED with TBN-TPA depended on the increasing concentration of TBN-TPA. Under the same voltage, the s-OLED with higher concentrations of TBN-TPA as emitter showed lower current density (shown in Figure 2A). In contrast, the current efficiency (CE), power efficiency (PE), and EQE increased as the doping concentration increased, shown in Figure 2B and Table 1. The device with 20 wt% TBN-TPA depicted the maximum EQEs of 1.08% with narrow FWHM of 32 nm. The performances of OLEDs with solution-processed TBN-TPA as blue TADF emitter are much lower than those of the devices with physical vapor deposition, which may be ascribed to the unsatisfactory film quality fabricated by spin-coating (Liang et al., 2018; Tang et al., 2020). Solution-processed organic thin films are generally of a lower quality than those fabricated by physical vapor deposition. More morphological defects could be induced and thus deteriorate charge transport properties and radiative decays in the solution-processed devices. Therefore, it is reasonable that solution-processed OLEDs generally exhibit lower EQEs compared with that of vapor deposition OLEDs using the same emitter despite the simple fabrication processing (Diao et al., 2014). Therefore, more effort should be devoted to device engineering.

**FIGURE 3:**
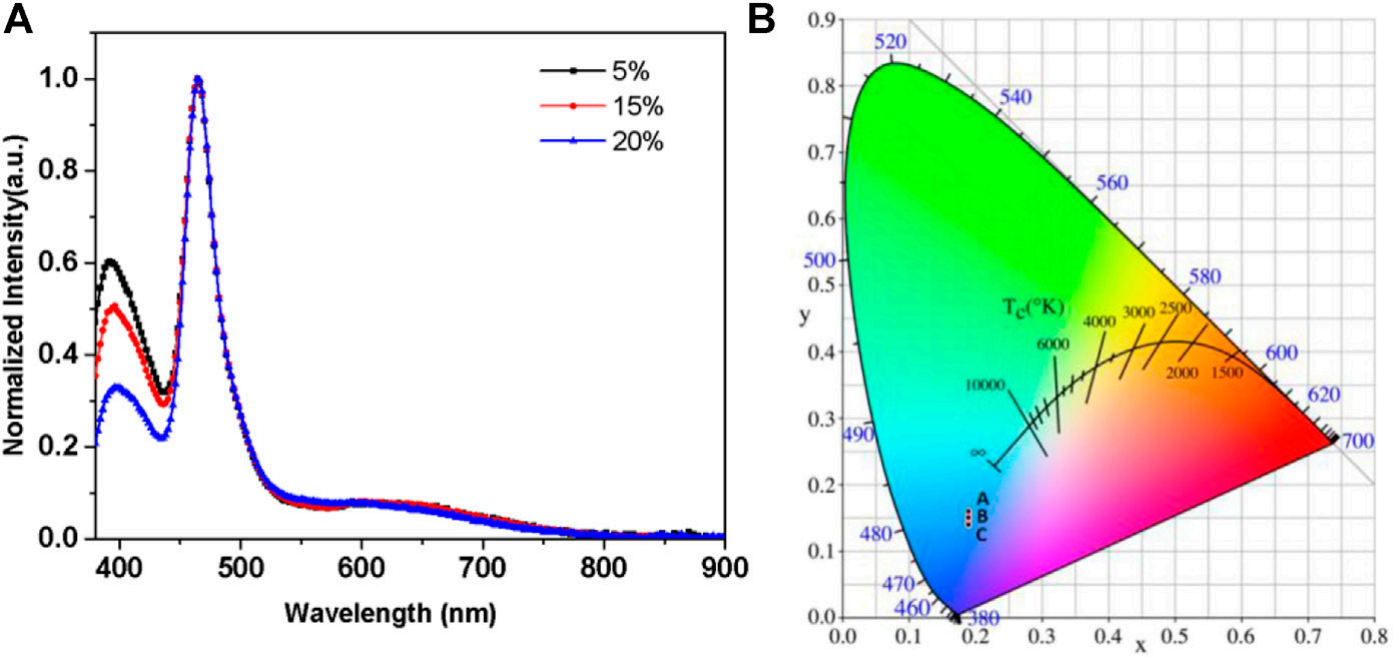
**(A)** The normalized EL spectra of devices and **(B)** the chromaticity coordinates of devices.

The EL spectrum and the chromaticity coordinates of the devices with TBN-TPA are shown in Figures 3A,B. The two peaks of the normalized EL spectra of the devices could be determined, which originated from the blue TADF emitter TBN-TPA and host mCP, respectively, peaking at 464 and 396 nm (shown in Figure 3A). This is attributed to the inefficient host-guest energy transfer. As for the chromaticity coordinates of the devices, the CIE (x,y) coordinates were slightly changed with the y value below 0.2 in Figure 3B. As the concentration of TBN-TPA increased from 5 to 20%, the residual emission of the host mCP was gradually quenched by TBN-TPA, which resulted in relatively higher EQEs, shown in Table 1. Meanwhile, there exists some interfacial exciplex which accounts for the emission band in the region of 600–700 nm.

## Conclusion

In summary, we succeeded in employing blue TADF dye with the narrow bandwidth in solution-processed OLED as the emitter to realize high color purity. This technical route shows high value in the development of solution-processed OLED display technology.

## Data Availability

The raw data supporting the conclusions of this article will be made available by the authors, without undue reservation.
